# PRAGMATIC CLINICAL TRIALS TO IMPROVE DEMENTIA CARE: IMPLEMENTATION, ADAPTATION, AND OUTCOMES

**DOI:** 10.1093/geroni/igad104.1636

**Published:** 2023-12-21

**Authors:** Kali Thomas, Joseph Gaugler

## Abstract

Pragmatic clinical trials test the effectiveness of interventions in “real-world” settings. One challenge with conducting such research in non-controlled environments is exposure to unpredictable events: in the case of the studies here, the COVID-19 pandemic. This session shares the implementation experiences and findings from four pragmatic clinical trials designed to improve dementia care and describes how the studies were impacted by and adapted to respond to the pandemic. The first speaker discusses a successful, team-based approach for adapting and implementing an intervention to improve sleep for nursing home residents with dementia. The second speaker presents the modifications made to implementation strategies between two trials of a personalized music intervention delivered to nursing home residents with dementia. The third speaker discusses findings from a mixed-methods evaluation with frontline staff at VA Community Living Centers enrolled in a stepped-wedge cluster randomized trial of Montessori-based approaches to improve dementia care. The fourth speaker shares findings from a pilot trial comparing two modes of meal delivery to homebound older adults with dementia. The discussant situates these adaptations and findings in the context of lessons learned by other trialists working to improve care provided to people living with dementia.

